# Prevention or Early Cure of Type 1 Diabetes by Intranasal Administration of Gliadin in NOD Mice

**DOI:** 10.1371/journal.pone.0094530

**Published:** 2014-04-11

**Authors:** David P. Funda, Petra Fundova, Axel Kornerup Hansen, Karsten Buschard

**Affiliations:** 1 The Bartholin Instituttet, Rigshospitalet, Copenhagen, Denmark; 2 Department of Immunology and Gnotobiology, Institute of Microbiology, v.v.i., Academy of Sciences of the Czech Republic, Prague, Czech Republic; 3 ENT Department of the 3rd Faculty of Medicine, Charles University and the Central Military Hospital, Prague, Czech Republic; 4 Section of Biomedicine, Department of Veterinary Disease Biology, Faculty of Health and Medical Sciences, University of Copenhagen, Frederiksberg, Denmark; La Jolla Institute for Allergy and Immunology, United States of America

## Abstract

Induction of long-term tolerance to β-cell autoantigens has been investigated both in animal models and in human type 1 diabetes (T1D) in order to prevent the disease. As regards external compounds, the dietary plant protein fraction has been associated with high penetrance of the disease, whereas gluten-free diets prevent T1D in animal models. Herewith we investigated whether intranasal (i.n.) administration of gliadin or gluten may arrest the diabetogenic process. I.n. administration of gliadin to 4-week-old NOD mice significantly reduced the diabetes incidence. Similarly, the insulitis was lowered. Intranasal gliadin also rescued a fraction of prediabetic 13-week-old NOD mice from progressing to clinical onset of diabetes compared to OVA-treated controls. Vaccination with i.n. gliadin led to an induction of CD4^+^Foxp3^+^ T cells and even more significant induction of γδ T cells in mucosal, but not in non-mucosal lymphoid compartments. This prevention strategy was characterized by an increased proportion of IL-10 and a decreased proportion of IL-2, IL-4 and IFN-γ-positive CD4^+^Foxp3^+^ T cells, and IFN-γ-positive γδ T cells, preferentially in mucosal lymphoid organs. In conclusion, i.n. vaccination with gliadin, an environmental antigen with possible etiological influence in T1D, may represent a novel, safer strategy for prevention or even early cure of T1D.

## Introduction

The incidence of type 1 diabetes mellitus (T1D) has been rapidly increasing during the past decades [1]. In humans, the autoimmune process is prolonged as clinical onset of the disease does not occur until approximately 80% or more β cells are destroyed [2] leaving a time window of opportunity for therapeutical or preventive intervention in prediabetic individuals. In animal models of T1D, mucosal administration of β-cell related autoantigens is a well-established strategy for disease prevention by induction of islet-specific T regulatory cells (Tregs) that may prevent the autoimmune aggression locally by mechanism of bystander suppression [3]. Several β-cell autoantigens, defined rather by occurrence of autoantibodies than T cell specific immunoreactivity exclusive for T1D patients, have been used for prevention of T1D by mucosal (oral, intranasal) administration. Mucosal administration of insulin [4], [5] or GAD [6], GAD65 peptide [7] as well as proinsulin or insulin peptides [8], [9] has led to prevention of T1D in animal models. Several of the autoantigens have been used in recent human trials, but at present, there is no established prevention strategy available for human T1D [10], [11].

Studies in both NOD mice and BB rats have documented that T1D is a diet-influenced disease. Wheat flour is an essential component of diabetes-permissive, non-purified diets and purified diets based on hydrolyzed casein, lactalbumin or amino acids prevented development of diabetes in NOD mice and BB rats [12]–[14]. We and others have documented that a non-purified, gluten-free diet highly prevents development of diabetes in NOD mice [15], [16].

The diabetogenic role of gliadin is also implicated by a more frequent clinical association of T1D and celiac disease. Although celiac disease and T1D share similar risk HLA antigens, patients diagnosed with both celiac disease and T1D usually develop diabetes first and not vice versa [17]. Patients with celiac disease have an earlier onset of T1D [18] and there is also one report of enhanced T reactivity to gluten in newly diagnosed type 1 diabetics [19]. Early introduction of dietary gluten was reported to increase the risk of developing islet autoantibodies in children [20]. Gluten-free diet also induces changes in the gut microbiota of NOD mice [21]. These observations suggest an etiological role for gliadin in T1D.

Using the NOD mouse model, we investigated the effect of intranasal (i.n.) administration of gluten or gliadin at the age of 4 weeks on development of insulitis and clinical onset of diabetes. We tested whether i.n. vaccination may rescue animals from developing diabetes when applied just before clinical onset of diabetes at the age of 13 weeks. We also investigated whether this i.n. prevention strategy leads to induction of potentially regulatory T cells and changes in their cytokine profiles.

## Methods

### Ethics statement

All animal experiments were carried out according to the principles of the EU directive 86/609, NIH publication no. 85–23 (revised 1985), and the national animal experimentation act. The study was approved by the National Animal Experimentation Board under the Danish Government Ministry of Food Affairs according to EU directive 86/609, license number 2007/561−1434-C3.

### Animals

NOD/BomTac mice were obtained from Taconic Europe A/S, Ry, Denmark and kept under barrier-protected conditions according to the FELASA guidelines [22]. The mice had free access to acidified drinking water and were fed standard Altromin 1324 diet (Altromin, Lage, Germany).

### Reagents and antibodies

Crude wheat gluten and ovalbumin (OVA) were obtained from Sigma (Sigma, St. Louis, MO), while gliadin was from Fluka (Sigma). Phorbol myristate acetate (PMA) and ionomycin were purchased from Sigma. The following monoclonal antibodies (mAbs) as well as isotype controls were purchased from BD Biosciences (BD Biosciences, Mountain View, CA): Alexa Fluor 488-conjugated rat anti-mouse IL-2 (JES6-5H4, IgG_2b_), IL-4 (11B11, IgG_1_), IFN-γ (XMG1.2, IgG_1_), FITC-conjugated rat anti-mouse IL-10 (JES5-16E3, IgG_2b_) and CD8 (53-6.7; IgG2a,κ), PerCP-Cy5.5-conjugated hamster anti-mouse CD3 (145-2C11; IgG1,κ), rat anti-mouse CD4 (RM4-5; IgG2a,κ), CD8 (53-6.7; IgG2a,κ) and PE-conjugated hamster anti-mouse γδ T cell receptor (GL3; IgG2,κ) mAbs. Mouse Treg staining kit Cat.No. 88–8111, PE-conjugated rat anti-mouse Foxp3 mAb (FJK-16s; IgG2a,κ) and FITC-conjugated rat anti-mouse CD4 mAb (RM4-5; IgG2a,κ) were from eBioscience (eBioscience, San Diego, CA). The anti-mouse CD4 mAb (BD Biosciences) was used in combination with intracellular cytokine staining using Cytofix/Cytoperm kit (BD Biosciences), while the anti-mouse CD4 mAb (from the eBioscience kit no. 88–8111) was used when detecting Foxp3^+^CD4^+^ T cells (with no prior PMA inomycin stimulation) by using the Treg staining kit 88–8111.

### Intranasal immunization and monitoring of diabetes

Non-anesthetized 4-week-old NOD female mice (n = 16 per group) were intranasally (i.n.) given 50 µg of OVA, gliadin, and/or gluten in a total volume of 10 µl (5 µl per nostril). Gliadin, gluten as well as OVA were dissolved in acidified (0.2% acetic acid) saline solution. Animals were immunized 5 times every other day. Five animals per group at the age of 13 weeks were used in separate experiments for insulitis scoring. NOD mice kept in our facilities start to progress to clinical onset of diabetes (>12 mmol) at the age of 14–15 weeks. Thus, at the age of 13 weeks these NOD mice have most of their islets affected by various stages of the mononuclear infiltrate, i.e. autoimmune aggression against β-cells. In order to test whether i.n. gliadin vaccination may prevent diabetes also in animals with advanced autoimmune reaction against β-cells, i.n. gliadin was tested in 13-week-old prediabetic NOD mice (n = 20 per group). For diabetes incidence studies, 16 to 20 mice per group were monitored for 210–240 days. NOD mice were inspected daily for diabetes and from 10 weeks of age screened weekly for glycemia with Glucometer FreeStyle mini (Hermedico, Brøndby, Denmark). Diagnosis of diabetes was based on two consecutive positive blood glucose readings >12 mmol/l during three days.

### Histology and insulitis scoring

Insulitis scoring was performed on hematoxylin & eosin stained pancreata from non-diabetic NOD females (n = 5) at the age of 13 weeks; the age at which non of the animals progressed to clinical onset of diabetes in our SPF animal facility, while in control groups majority of islets were affected by some presence of mononuclear infiltrate (insulitis grades 1–4). One half of the pancreata was fixed in 4% formaldehyde, embedded in paraffin, cut in 5 µm sections and stained with hematoxylin & eosin for insulitis scoring. The grades for insulitis scoring were as follows: 0, normal islet; 1, intact islet with few scattered mononuclear cells in the surroundings; 2, peri-insulitis; 3, insulitis (<50% of the islet infiltrated); 4, severe insulitis (>50% of the islet infiltrated). A minimum of 25 islets were scored for each mouse and this experiment was carried out independently of diabetes incidence studies. Data are expressed as average percentage of islets affected by the 5 (0–4) insulitis grades (percent of islets) calculated from 5 animals per group.

### Flow cytometry

Single cell suspensions of 8-week-old, normoglycaemic animals were prepared from the following mucosal lymphoid tissues: the nasal-associated lymphoid tissue (NALT), pancreatic lymph nodes (PLN), and mesenteric lymph nodes (MLN). Spleens (S) and systemic (inguinal) lymph nodes (ILN) were used as non-mucosal controls. For detection of Foxp3^+^CD4^+^ Tregs, intracellular staining for Foxp3 was carried out using the Treg staining kit 88–8111 (eBioscience) following the manufacturer procedure. When detecting intracellular cytokines, isolated cells were stimulated in vitro with a mixture of PMA (25 ng/ml) and ionomycin (1 µg/ml) in RPMI-1640, 10% FCS for 4 hours at 37°C, 5% CO_2_ in the presence of Golgi Stop (Cytofix/Cytoperm kit, BD Biosciences) before subsequent staining for selected surface and intracellular markers. Titration experiments were performed to determine optimal lengths (1–6 hours) and concentrations of PMA/ionomycin stimulation. Unstimulated cells cultured in the presence of Golgi Stop were used as controls. For surface staining, cells were incubated in FACS buffer with relevant mAbs for 30 minutes on ice. Fc block (CD16/CD32) was from BD Biosciences. For intracellular staining of cytokines, live cells were first stained for surface markers, then fixed/permeabilized with the Cytofix/Cytoperm kit following the manufacturer procedure. No difference in the staining was observed when comparing sequential versus one step procedure. Thus, anti-Foxp3 and an anti-cytokine mAbs were added in a single incubation step. Cells from OVA- and gliadin-treated mice were prepared, stained and measured on the same day. Cells were then analyzed by flow cytometry using a FACSscan (BD Biosciences), and data were analyzed by use of CellQuest (BD Biosciences), WinMDI 2.8 and/or FlowJo (TreeStar) software. Only very few cytokine-positive cells were detected in unstimulated controls. Isotype control antibodies were used to determine the amount of non-specific binding, and propidium iodide was used to localize and assess proportion of dead cells prior their fixation/permeabilization.

### Statistics

The cumulative diabetes incidence was assessed using the Kaplan-Meier estimation and contingency tables. Log-rank test and Chi-squre test were used for comparisons between groups. Other results are expressed as mean ±SEM, and the level of significance (p<0.05) was assayed by two-sample analysis (unpaired t-test) or ANOVA followed by the Bonferroni multiple comparison test (comparison of multiple groups in insulitis scoring).

## Results

### I.n. administration of gliadin leads to reduced diabetes incidence in NOD mice

**Figure 1:**
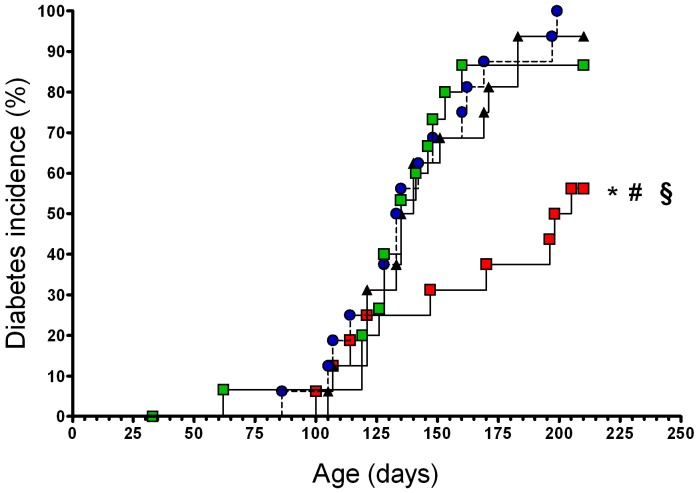
Intranasal (i.n.) administration of gliadin decreases diabetes incidence in NOD mice. A statistically significant decrease in diabetes incidence was found in NOD mice treated with gliadin (red square) compared to OVA (blue circle), *p = 0.001 or PBS (black triangle), #p = 0.008 controls, whereas no diabetes-protective effect was found in gluten-treated (green square) group. The gliadin-treated group displayed decreased diabetes incidence (§p = 0.029) also compared to gluten-treated (closed circle) NOD mice. Results are representative of three or two (gluten) independent experiments.

As shown in Fig. 1., five intranasal administrations of gliadin (50 µg) to 4-week-old NOD females significantly decreased the diabetes incidence to 56% in comparison to mice treated with OVA with a diabetes incidence of 100%, p = 0.001 (by log-rank test) and to PBS-treated controls (94%, p = 0.008). There were no substantial differences in the development of diabetes incidence between the phosphate-buffered saline (PBS) and OVA-treated control groups. I.n. administered wheat gluten consisting mainly of glutenins and gliadins and thus comprising the gliadin fraction, although in smaller amounts, had no effect on the diabetes incidence (81% at 210 days) compared to PBS and OVA controls (Fig. 1). The difference in diabetes incidence between the gluten (81%) and gliadin (56%) treated groups was again statistically significant at p = 0.029 (by log-rank test). Additionally, we investigated a more intense i.n. application scheme in which the five intranasal application of gliadin (50 µg) every other day were first applied to 4-week-old NOD mice and then repeated 2 times more, each time with a 10-day break interval. However, this high-frequency scheme of i.n. gliadin resulted in no significant diabetes prevention in NOD mice (data not shown) and is in accord with data from a mathematical model of NOD mice published by Fousteri G. et al. [23] in which high-frequency i.n. immunizations also failed in simulated disease protection. We also tested whether i.n. administration of gliadin leads to induction of tolerance but found no differences among the gliadin, OVA (and PBS) i.n. treated groups of mice in serum anti-gliadin IgG after s.c. immunization with gliadin in CFA as well as in cytokine recall responses (IFN-γ IL-5 and IL-10) after in vitro restimulation of MLNs suspensions (data not shown).

### Reduction of insulitis by i.n. administration of gliadin

**Figure 2:**
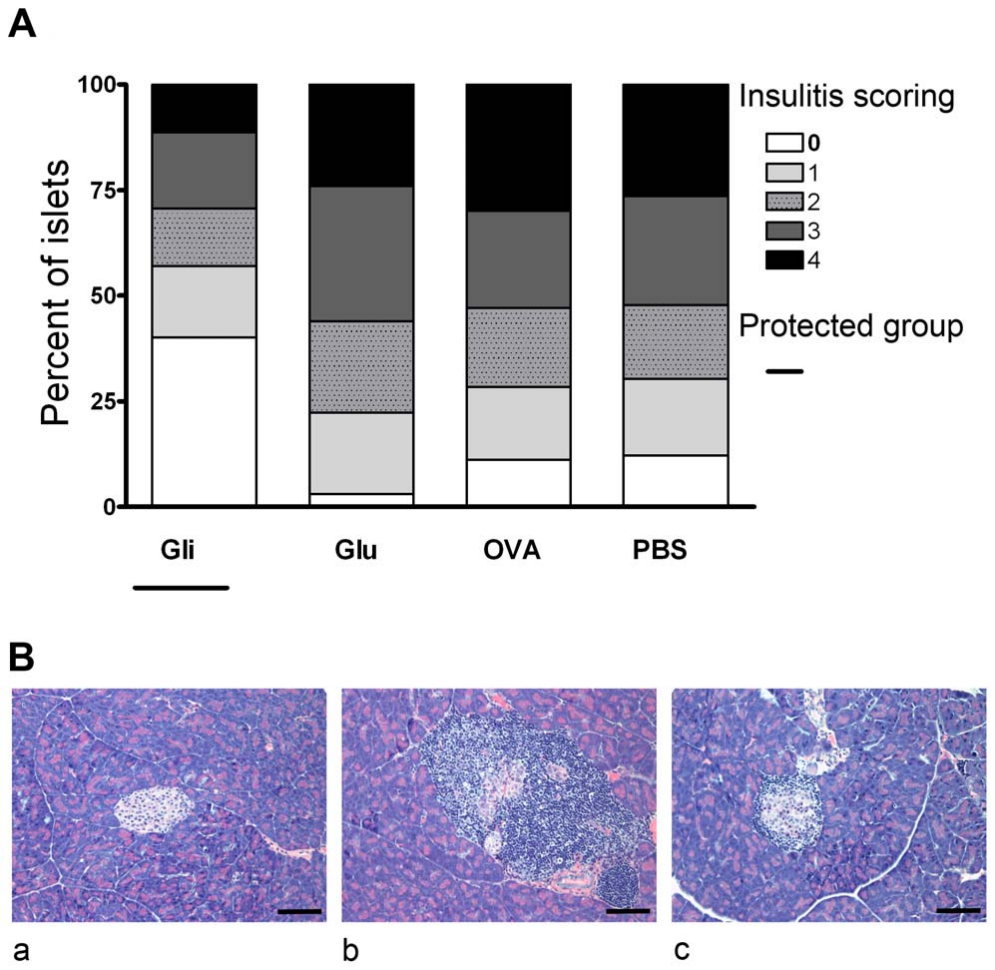
Intranasal administration of gliadin but not gluten prevented development of insulitis in NOD mice. (A) Insulitis was assessed by histological examination at 13 weeks of age before clinical onset of diabetes. The five grades (0–4) for insulitis scoring are described in the Research Design and Methods. Five mice per group and a minimum of 25 islets/mouse were scored and this experiment was carried out independently of diabetes incidence studies. (B) Photomicrographs of H&E stained histological specimens documenting an example islet of grade level 0 (a), 4 (b) and 3 (c) used for insulitis scoring. Example photomicrographs of grade level 0 (a), 4 (b) and 3 (c) are from 13-week-old NOD mice treated with gliadin, OVA, and gluten, respectively. Scale bars: 100 µm.

Insulitis scores from 13-week-old NOD female mice are overviewed in Fig. 2. Gliadin-treated NOD females (insulitis score 1.44±0.11) revealed statistically significant, less destructive insulitis compared to OVA (2.44±0.11, p<0.001) and PBS (2.36±0.10; p<0.001), but also to gluten (2.55±0.09; p<0.05) treated groups (Fig 2A). There were no significant differences among the gluten-treated and control (PBS, OVA) groups. Thus, in accord with the diabetes incidence data, insulitis scoring indicated a significant beneficial effect of gliadin on islet preservation. Animals used for insulitis scoring were from time different i.n. experiment. Fig. 2B illustrates examples of three different grades used for insulitis scoring.

### I.n. gliadin reduces diabetes incidence even in 13-week-old NOD mice just before the clinical onset of diabetes

**Figure 3:**
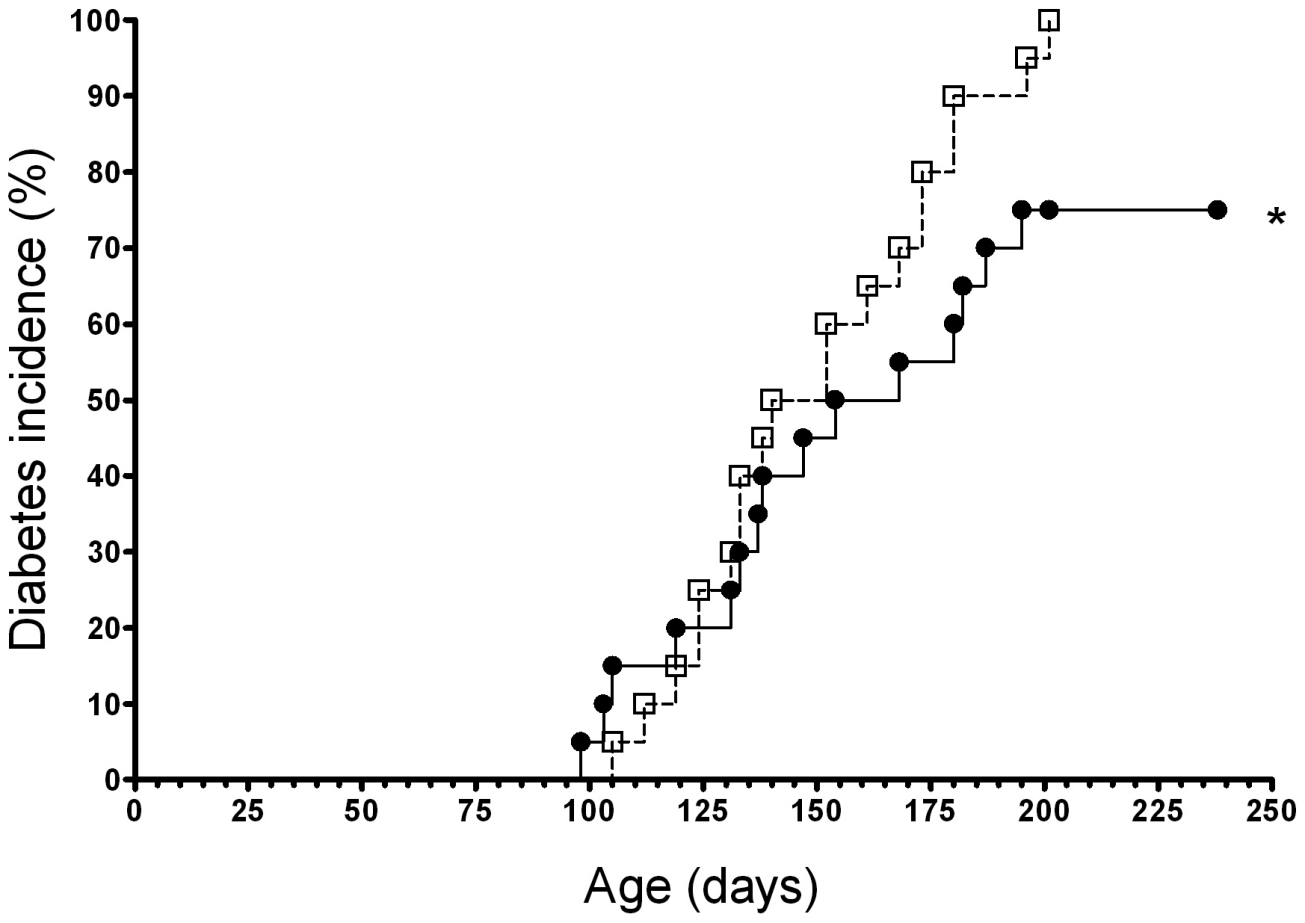
Intranasal administration of gliadin in prediabetic NOD mice with advanced insulitis decreases diabetes incidence. Groups (n = 20) of 13-week-old NOD female mice received five i.n. administrations (every other day). A statistically significant decrease in diabetes incidence was found at the age of 240 days in prediabetic NOD mice treated with gliadin (closed circle) compared to OVA control (opened square); *p = 0.017. While control OVA-treated mice display 100% diabetes incidence, only 75% gliadin-treated littermates progressed to the disease at 240 days.

Five i.n. administrations of gliadin to prediabetic NOD females at the age of 13 weeks decreased the diabetes incidence compared to OVA-treated controls (p = 0.017, contingency tables and Chi-squre test, Fig. 3). Thus, i.n. administration of gliadin could rescue a small, but statistically still significant, proportion of the animals from progressing towards manifestation of the disease with glycemia values still below 12 mmol, in spite of a high degree of autoimmune infiltrate and damage present at this age in endocrine pancreata of NOD mice.

### I.n. administration of gliadin increases number CD4^+^Foxp3^+^ T cells in mucosal but not in non-mucosal lymphoid organs

**Figure 4:**
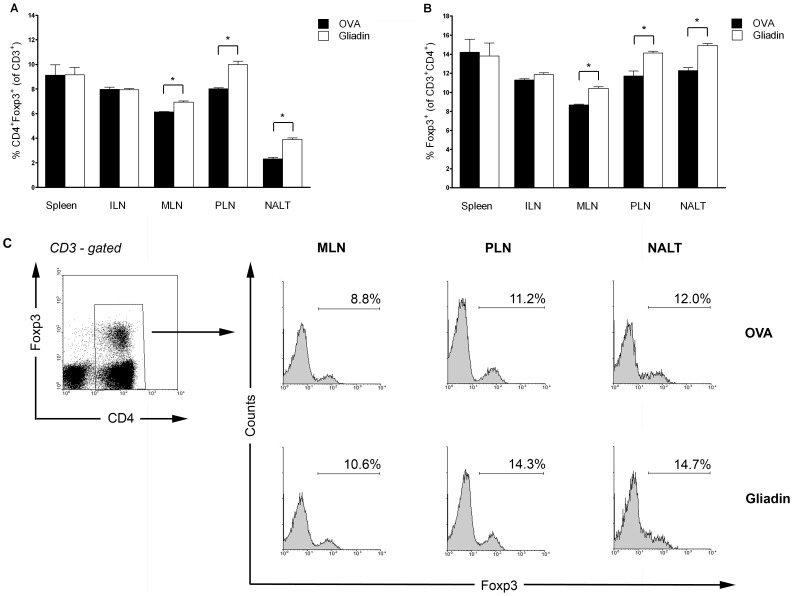
Induction of CD4^+^Foxp3^+^ T cells by intranasal administration of gliadin. (A) Proportion of CD4^+^Foxp3^+^ cells within T (CD3-gated) cells in mucosal (NALT, MLN, PLN) and non-mucosal compartments (Spleen, ILN) after i.n. gliadin (white bars) or OVA (black bars) vaccination in 8-week-old NOD female mice. (B) Proportion of CD4^+^Foxp3^+^ cells expressed as percentage of CD3^+^CD4^+^ T cell subset; lymphoid organs and bars as ad A. (C) Example FACS analysis of CD4^+^Foxp3^+^ T cells in MLN, PLN and NALT of gliadin and OVA-treated NOD mice. Cells were gated according to the CD3 parameter and Foxp3 expression analyzed within the CD4-positive cells (histograms). Individual measurements were performed on cells pooled from 2-3 experimental animals. Data are expressed as mean values ±SEM and represent an example of two independent experiments. *p<0.05.

I.n. administration of gliadin to NOD mice led to increased number of CD4^+^Foxp3^+^ T cells at 8 weeks of age. Increased proportion of these cells was gliadin-specific in comparison with the i.n. administered control protein - OVA at the site of the antigen administration - in the NALT and in the mucosal draining lymph nodes of the pancreas – PLN, and MLN. Thus, an increase of CD3^+^CD4^+^Foxp3^+^ cells was found in NALT (p = 0.013), MLN (p = 0.014) and PLN (p = 0.019) after i.n. gliadin vaccination (Fig. 4A). These data are significant also when expressed as proportion of CD4^+^ helper T cells (NALT, p = 0.019; MLN, p = 0.011; PLN, p = 0.049), (Fig. 4B and C). Interestingly, the i.n. gliadin-mediated increase of Foxp3^+^ T cells was not found in other non-mucosal lymphoid organs such as spleen and control systemic ILN (Fig. 4).

### I.n. administration of gliadin leads to increased numbers of γδ T cells in mucosal but not in non-mucosal lymphoid organs

**Figure 5:**
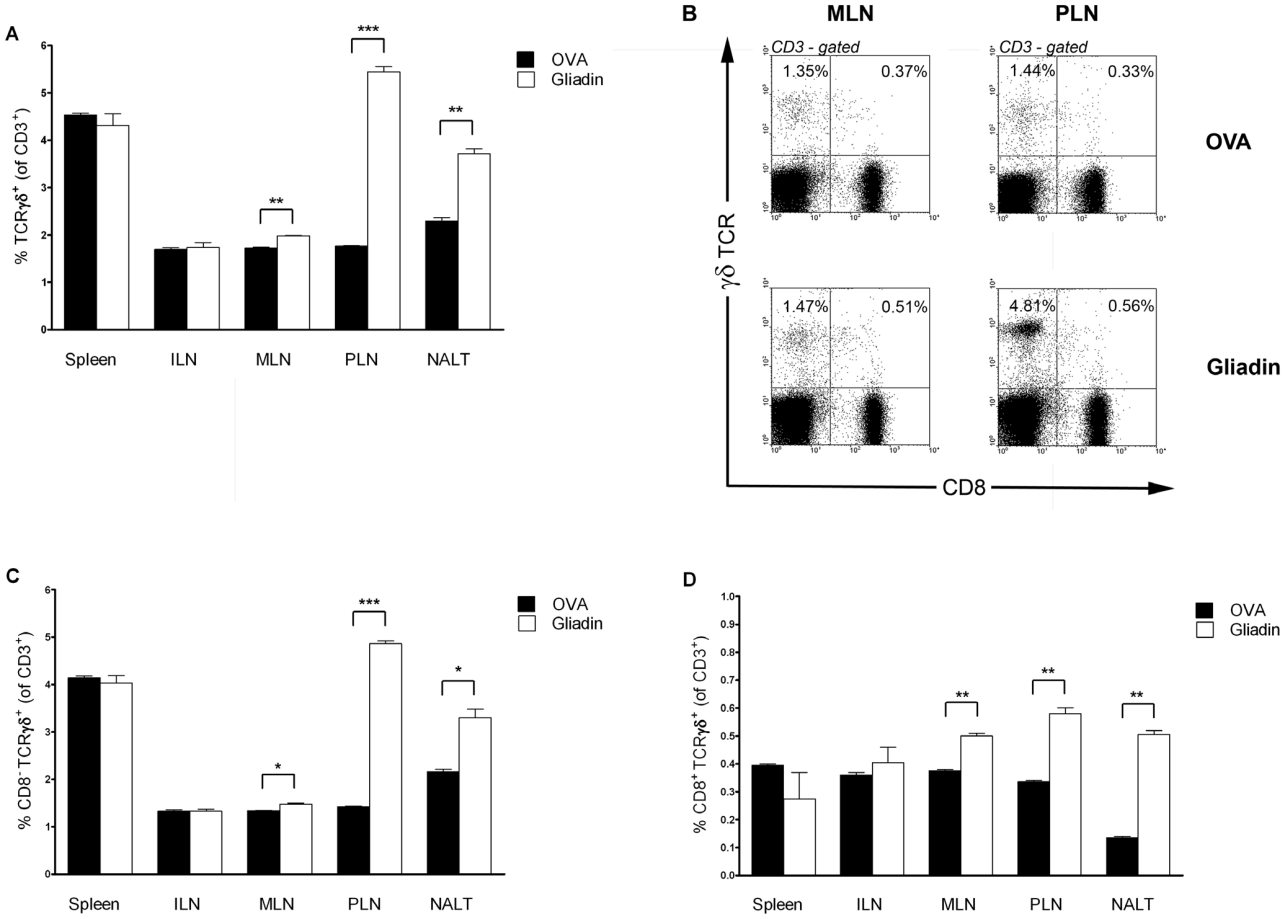
Effect of i.n. administration of gliadin on frequency of γδ T cells. (A) Proportion of γδ T cells within T (CD3-gated) cells in mucosal (NALT, MLN, PLN) and non-mucosal compartments (Spleen, ILN) after i.n. gliadin (white bars) or OVA (black bars) administration in 8-week-old NOD female mice. Panels C and D show further analysis of the γδ T cells according to their CD8 expression; lymphoid organs and bars as ad A. (C) Proportion of CD8^-^ γδ T cells expressed as percentage of CD3^+^ T cells. (D) Proportion of CD8^+^ γδ T cells expressed as percentage of CD3^+^ T cells. (B) Example FACS analysis of γδ T cells (CD3-gated) in MLN and PLN of NOD mice treated i.n. with gliadin or OVA. Individual measurements were performed on cells pooled from 2–3 experimental animals. Data are expressed as mean values ±SEM and represent an example of two independent experiments. *p<0.05, **p<0.01, ***p<0.001.

I.n. administration of gliadin to NOD mice led to a mucosal-specific accumulation of γδ T (CD3^+^) cells at the age of 8 weeks. Thus i.n. gliadin led to a substantially increased frequency of γδ T cells in NALT (p = 0.004), MLN (p = 0.004), and PLN (p = 0.001), but not in non-mucosal lymphoid organs such as spleen and ILN compared to OVA-treated controls (Fig. 5A and B). The increased frequency of γδ T (CD3-gated) cells was found both within the CD8^-^ γδ T cell subset (NALT, p = 0.027; MLN, p = 0.013; PLN, p = 0.001) as well as in γδ T cells expressing CD8 marker (NALT, p = 0.002; MLN, p = 0.008; PLN, p = 0.007) (Fig. 5C and D). In gliadin-treated NOD mice, there was a shift towards increased proportion of CD8^+^ γδ T (CD3^+^) cells at the site of the immunization - the NALT and MLN. On the other hand, a relatively higher proportion of CD8^-^ γδ T (CD3^+^) cells was detected in the draining lymph nodes of pancreas - the PLN (Fig. 5C and D).

### Cytokine profiles of CD4^+^Foxp3^+^ and γδ T cells after i.n. administration of gliadin in mucosal and non-mucosal lymphoid organs

**Figure 6:**
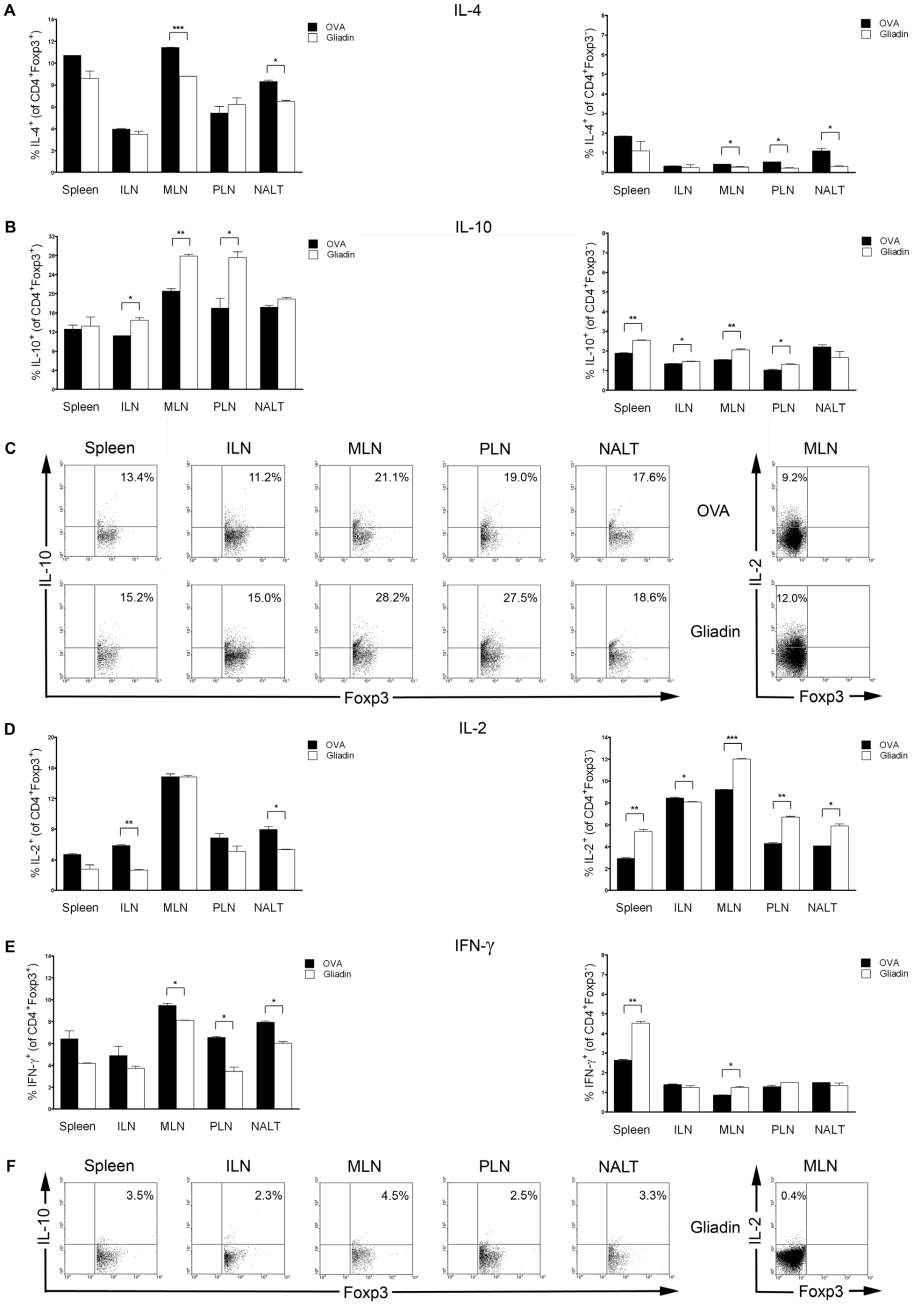
Cytokine profiles of CD4^+^Foxp3^+^ and CD4^+^Foxp3^-^ cells after i.n. administration of gliadin. Frequency of cytokine-positive cells in mucosal (NALT, MLN, PLN) and non-mucosal compartments (Spleen, ILN) after 4 hours unspecific in vitro restimulation with PMA/ionomycin is shown in panel A (IL-4), B (IL-10), D (IL-2) and E (IFN-γ). Left panels display cytokines expression in CD4^+^Foxp3^+^ and right panels in CD4^+^Foxp3^−^ cells. (C) Example FACS analysis of IL-10 expression within CD4^+^Foxp3^+^ (Spleen, ILN, MLN, PLN and NALT) and IL-2 positive cells within CD4^+^Foxp3^−^ subset (MLN) of 8-week-old NOD mice vaccinated i.n. with gliadin or OVA at 4 weeks of age. (F) Example of IL-10 and IL-2 positive cells in unstimulated controls (cell subsets and organs as at C). Individual measurements were performed on cells pooled from 2–3 experimental animals at age of 8 weeks. Data are expressed as mean values ±SEM, i.n. gliadin (white bars), i.n. OVA (black bars). *p<0.05, **p<0.01, ***p<0.001.

I.n. administration of gliadin is associated with increased amount of IL-10 and decreased amount of IL-2, IL-4 and IFN-γ in CD4^+^Foxp3^+^ T cells, preferentially in mucosal lymphoid organs in 8-week-old NOD mice. Following PMA/ionomycin stimulation in vitro, an increased number of IL-10 positive CD4^+^Foxp3^+^ cells was found both in MLN (p<0.01) and PLN (p<0.05), but also in non-mucosal ILN (p<0.05) of the gliadin-treated NOD mice at the age of 8 weeks (Fig. 6B and C). On the other hand, CD4^+^Foxp3^+^ T cells from the gliadin-treated group displayed lower potential to produce IL-4 (MLN, p<0.001; NALT, p<0.05), IL-2 (ILN, p<0.01; NALT, p<0.05), and IFN-γ (MLN, p<0.05; PLN, p<0.05; NALT, p<0.05) (Fig. 6A, D and E). Only very few cytokine-positive cells were detected in unstimulated controls (example data in Fig. 6F). The most consistent difference was observed in the IFN-γ production of CD4^+^Foxp3^+^ T cells that was significantly reduced in all the mucosal lymphoid organs studied, including the pancreatic draining PLNs (Fig. 6E).

Within the CD4^+^Foxp3^−^ subset of T cells, only a small percentage of cells was positive for IL-4, IL-10 and IFN-γ. Substantially more CD4^+^Foxp3^−^ T helper cells were positive for IL-2 and a significantly increased number of IL-2 producing CD4^+^Foxp3^−^ T helper cells was detected in all organs studied (Fig. 6D). IL-10 positive CD4^+^Foxp3^−^ T cells were also more frequent in all studied organs except for NALT in the gliadin-treated group (Fig. 6B and C). A decreased amount of IL-4 was found in all mucosal (MLN, p<0.05; PLN, p<0.01; NALT, p<0.05) lymphoid tissues of gliadin-treated mice, whereas IFN-γ was increased in spleen (p<0.01) and MLN (p<0.05), (Fig. 6A and E).

**Figure 7:**
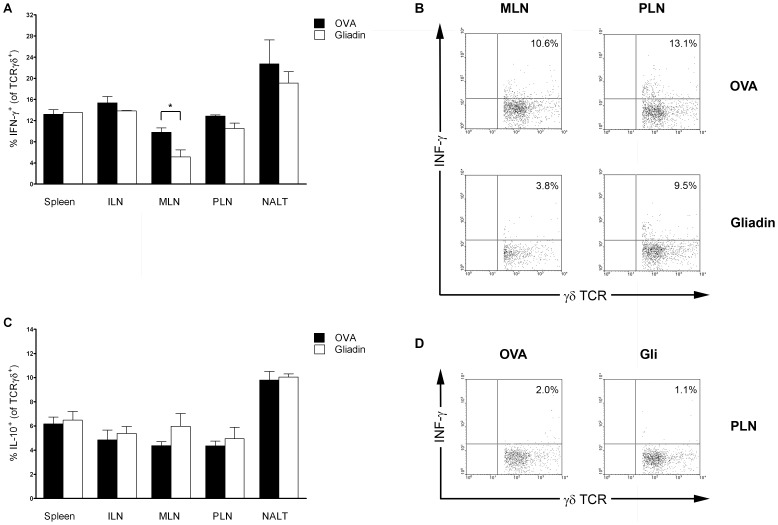
Expression of IL-10 and IFN-γ in γδ T cells after i.n. administration of gliadin. Proportion of IFN-γ (panel A) and IL-10 (panel C) positive γδ T cells within mucosal (NALT, MLN, PLN) and non-mucosal compartments (Spleen, ILN) after i.n. gliadin (white bars) or OVA (black bars) administration in 8-week-old NOD female mice. (B) Example FACS analysis of IFN-γ expression within γδ T cells in MLN and PLN of NOD mice treated i.n. with gliadin or OVA after 4 hours in vitro restimulation with PMA/ionomycin. (C) Example of IFN-γ positivity within γδ T cells of unstimulated controls (PLN) from gliadin-treated NOD mice. Individual measurements were performed on cells pooled from 2–3 experimental animals. Data are expressed as mean values ±SEM, *p<0.05.

Almost no IL-2 and IL-4 positive γδ T cells were detected after in vitro restimulation with PMA and ionomycin (data not shown) regardless of the i.n. treatment. Although i.n. gliadin led to significantly increased induction of γδ T (CD3^+^) cells in all studied mucosal lymphoid organs (Fig. 5), γδ T cells displayed no substantial differences in positivity for IL-10 as regards the i.n. treatment with gliadin and OVA (Fig. 7C). Diabetes-preventive i.n. administration of gliadin was associated with a decreased number of IFN-γ positive γδ T cells in MLN (p<0.05) and a similar tendency was observed in their PLN (Fig. 7A and B).

## Discussion

We have shown that five intranasal administrations of 50 µg gliadin in 4-week-old NOD mice significantly reduce penetrance of diabetes as well as the level of insulitis. This gliadin treatment was accompanied by an increase of CD4^+^Foxp3 T cells and much higher increase of γδ T cells in mucosal lymphoid compartments. Moreover, i.n. gliadin can even rescue a small fraction of prediabetic 13-week-old NOD mice (with a high degree of insulitis) from progressing to clinical onset of the disease. Thus, in this study an environmental antigen, closely related to the development of T1D, has been successfully applied in the disease prevention.

Prevention of T1D in NOD mice by i.n. gliadin was associated with an increased proportion of CD4^+^Foxp3^+^ and even more significant increase of γδ T cells specifically in the mucosal lymphoid compartments, but not in systemic lymphoid organs such as spleen and ILN (Fig. 4 and 5). Thus, these cells were found at the site of the i.n. application (NALT) at the draining lymph nodes (PLN) of the target organ, the pancreas, as well as in gut draining mucosal lymph nodes, MLN. This distribution pattern supports the concept of “common mucosal system” [24]. The increase of γδ T cells and Foxp3^+^ Tregs specifically in the pancreatic-draining PLN and also in MLN, in which priming of diabetogenic cells has been reported [25], points to possible mechanism of bystander suppression [26]. The increased proportion of IL-10 in Foxp3^+^ Tregs, preferentially within the mucosal compartment, and a decrease of IFN-γ in both Foxp3^+^ Tregs and γδ T cells after i.n. gliadin (Fig. 6 and 7) are in accord with the previously reported role of IL-10 cytokine produced by disease protective Tregs [5], [26], [27].

It has been suggested that a deficiency of Tregs could be associated with T1D development, and defective suppressor function in CD4^+^CD25^+^ T cells was reported in T1D patients [28], [29]. Although we found increased proportions of both Foxp3^+^ and γδ T cells after i.n. gliadin, the effect on γδ T cells seems to be substantially larger. γδ T cells are not generally considered as a typical Treg subset, however there are several lines of evidence for their involvement and even regulatory role in T1D [5], [30]–[32]. We have documented that NOD mice display an increased proportion of γδ T cells at onset of diabetes [33]. γδ T cells specific for insulin peptide B:9–23 were also reported in NOD mice [34]. On the other hand, γδ T cells play an important role in induction and maintenance of oral tolerance [35]. It has been shown that in the neonatal thymectomy NOD model of T1D, gut intraepithelial CD8^+^γδ T cells can prevent development of diabetes, and proper development of intraepithelial γδ T cells is required for induction of regulatory CD4^+^CD25^+^ T cells by oral insulin [32]. Interestingly, intranasal aerosol application of the whole insulin molecule in NOD mice led to induction of CD8^+^γδ T cells capable of preventing development of diabetes in an adoptive cotransfer model [5]. This study corresponds with our data documenting that i.n. administration of another whole-molecule antigen - gliadin - also led to preferential induction of γδ T cells.

Several of the animal-tested T1D autoantigens proceeded to human trials e.g. oral or intranasal insulin administration in humans at risk of type 1 diabetes (DPT-1, INIT). While some human trials are in progress, others such as the Diabetes Prevention Trial-1 (DPT-1) with oral or s.c. and i.v. insulin or s.c. GAD65/alum failed to show a protective effect [10], [36]–[38]. Also in other autoimmune diseases, human trials with oral autoantigens have not led to satisfactory outcomes - (reviewed in [39].

Nevertheless, there are a few aspects that may question the use of β-cell autoantigens in mucosal prevention of autoimmune diseases, in particular T1D. Firstly, type 1 diabetes is an autoimmune disease for which the Witebsky and Rose's original autoimmune criterion of disease-induction with a specific autoantigen has never been met [40]. In fact, immunizations with neither β-cell autoantigens nor pancreatic extract together with adjuvants were able to induce T1D, reviewed in [41]. Secondly, since mucosally administered antigens can induce both tolerance as well as immunity, any use of “self” antigens requires extensive testing and cautions for induction of autoimmunity [9], [42], [43]. Thus, vaccinations with β-cell related autoantigens have not so far proven valuable for humans, although combination therapy with immunosuppressive anti-CD3 mAb seems to be promising [11], [26].

In contrast to gliadin, i.n. administration of gluten did not lead to prevention of diabetes in NOD mice. This could be due to a lower dose of gliadin present in the gluten fraction. We hypothesize, it might also be a matter of availability - e.g. transports on mucosal surfaces and presentation by DCs, as glutenins are formed by much higher molecular weight polypeptide chains that tend to form a molecular net, thus may limit availability of various gliadins within the gluten fraction. The effect of gliadin on immune responses is not fully understood. Prevention of T1D by i.n. gliadin did not induced specific tolerance to gliadin. This might be due to the fact that the 4- or even 13-week-old NOD mice fed standard gluten-containing diet have already well-established oral tolerance to gliadin, since a very short period of oral antigen exposure is needed for induction of oral tolerance [44]. Gliadin induces activation of innate immune mechanisms and maturation of dendritic cells [45]. While gliadin-induced inflammatory cytokine production was described as MyD88-dependent, TLR2 and TLR4 were reported as not involved in the gliadin-induced signaling pathway [46], [47]. Furthermore, gliadin derived peptides may trigger T cell specific responses but also stimulate innate immunity response [48]. Gluten-free diets but also gluten content in diets influence diabetes incidence in animal models [15], [16], [49]. The etiological role of gliadin in T1D is supported by the study by Galipeau HJ et al. [50] documenting that while sensitization with gliadin induces only moderate enteropathy in humanized NOD-DQ8 mice, when combined with partial antibody depletion of Foxp3 Tregs it led to development of insulitis. The induction of mucosal γδ T cells by i.n. gliadin corresponds with our recent reciprocal finding: we found decreased proportions of γδ T cells in mucosal and non-mucosal compartments of BALB/c mice fed standard compared to a gluten-free diet [51].

The use of an external environmental substance, gliadin, in this study is novel and may have clinical implications. In addition, gliadin represents one of the most common food antigens in western countries, thus its safety in human application may be easier to test compared to β-cell related autoantigens [9], [42], [43].

In conclusion, our data show that i.n. mucosal application of gliadin is capable of significant prevention of diabetes and development of insulitis in NOD mice. In addition, i.n. gliadin displayed also small effect on preventing progression to clinical diabetes in just prediabetic animals with advanced autoimmune aggression within their islets. This prevention by an environmental antigen - gliadin - is associated with local, mucosal induction of γδ T cells and to much lesser extent CD4^+^Foxp3^+^ T cells. Because environmental factors play important roles in the recent increase of T1D, environmental antigens related to T1D should be considered for prevention trials. Our data suggest that intranasal, mucosal vaccination with gliadin may represent a novel and relatively safe approach to prevention and/or even early cure of type 1 diabetes.
